# Comparative efficacy of different exercise types on body composition in university students: a systematic review and meta-analysis of randomized controlled trials

**DOI:** 10.3389/fphys.2025.1537937

**Published:** 2025-04-22

**Authors:** Jihai Li, Liuhong Zang, Sihai Hao, Hui Wang

**Affiliations:** Institute of Physical Education, Xinjiang Normal University, Urumqi, Xinjiang, China

**Keywords:** obesity, body composition, BMI, exercise, dose, systematic evaluation, network meta-analysis

## Abstract

**Background:**

To systematically assess the impact of various exercise modalities and dosages on the body composition of college students through a comprehensive review of randomized controlled trials (RCTs).

**Methods:**

We conducted a comprehensive search of relevant randomized controlled trials (RCTs) in eight databases, covering data from the inception of each database to August 2024. Following the literature screening, two investigators independently conducted data extraction and assessed the risk of bias. Network meta-analysis (NMA) was conducted using Stata 17.0 with random-effects modeling, while dose-response analysis was performed utilizing R version 4.3.1.

**Results:**

A total of 43 randomized controlled trials (RCTs), encompassing 3,154 participants, were included in the analysis. Aerobic exercise, combined exercise, high-intensity interval training (HIIT), mind-body exercise, and calisthenics demonstrated significant effects on reducing body mass index (BMI) compared to control groups. Surface under the cumulative ranking (SUCRA) probability rankings indicated that calisthenics had the highest likelihood of being the most effective intervention for BMI reduction, whereas resistance exercise was associated with the lowest likelihood. The dose-response analysis revealed that the threshold exercise dose for overall exercise to lower BMI was 310 METs-min/week, with the predicted maximum significant response dose being 1,300 METs-min/week, beyond which there was minimal change in the intervention effect. Additionally, distinct nonlinear dose-response relationships were observed for aerobic exercise, combined exercise, HIIT, mind-body exercise, and aerobics.

**Conclusion:**

No significant differences in the effectiveness of exercise interventions on body composition were observed across exercise types. However, based on the SUCRA analysis, calisthenics emerged as the preferred intervention, succeeded by a combination of exercises. The optimal exercise dosage for enhancing body composition was identified as 1,300 METs-min/week, with the threshold for a significant effect being relatively low.

**Systematic Review Registration:**

https://www.crd.york.ac.uk/PROSPERO/view/CRD42024587032

## 1 Introduction

With shifts in lifestyle and dietary patterns, global obesity rates have increased threefold over the past 35 years (Bray et al., 2016), and projections indicate that by 2030, over 1 billion individuals will be classified as obese (WHO, 2024; Haslam and James, 2005). Obesity has emerged as a predominant health challenge worldwide, with strong correlations to an array of diseases, including cardiovascular, digestive, respiratory, musculoskeletal, depression, and anxiety (Haidar and Cosman, 2011; Kivimäki et al., 2022), obesity may also negatively affect an individual’s emotional intelligence (Gilyana et al., 2023). The direct and indirect economic costs attributable to obesity and its comorbidities are estimated to reach $2 trillion globally (González-Muniesa et al., 2017). The rapid advancement of digital technology has contributed to a sedentary lifestyle among college students, with more than 50% of students in the United States not meeting physical activity recommendations (Haase et al., 2004). A survey encompassing 23 countries revealed that a majority of college students in these nations engage in low levels of physical activity (Ezati et al., 2020). Concurrently, changes in dietary habits within university settings have contributed to a multifaceted set of factors driving the annual increase in obesity rates among college students (Lv et al., 2019; Vankim and Nelson, 2013).

Obesity interventions primarily encompass lifestyle modifications, dietary restrictions, augmented physical activity, and medical interventions, including pharmacotherapy and surgery (Bray et al., 2016). Exercise stands out as a straightforward and potent intervention, with various exercise modalities demonstrating efficacy in reducing body mass index (BMI), for example, a study by Batrakoulis et al. states that Combined training is the most effective type of exercise for improving obesity (Batrakoulis et al., 2022). However, there is a lack of consensus on the most effective type of exercise (Morze et al., 2021; O’Donoghue et al., 2021; Andreato et al., 2019; Wang et al., 2022; Wang H. et al., 2023; Hao et al., 2023), and scant research exists that scrutinizes the relationship between exercise dosage and BMI changes. Concurrently, college students, immersed in a communal campus environment and grappling with substantial academic demands (Meehan and Howells, 2019; Bayram and Bilgel, 2008), constitute a distinctive cohort within the adult population. Hence, we included pertinent randomized controlled trials (RCTs) focusing on college students and employed a synthesis of network meta-analysis and dose-response modeling to elucidate the optimal exercise type and dosage for obesity reduction in this demographic. This investigation aims to furnish college students with actionable insights for crafting personalized exercise regimens as part of obesity interventions.

## 2 Methods

### 2.1 Registration

This systematic review and NMA were conducted according to the Preferred Reporting Items for Systematic Reviews and Meta-Analyses statement guidelines (Hutton et al., 2015). The study protocol was registered in the International Prospective Register of Systematic Reviews ID: CRD42024587032.

### 2.2 Literature search strategy

Searches were conducted across five English-language databases—PubMed, Embase, Cochrane, Web of Science, and EBSCO—as well as three Chinese-language databases: CNKI, Wanfang, and VIP. For PubMed, Cochrane, and Embase, the search employed a blend of subject headings and keywords. The search strategy adhered to the PICOS framework, encompassing randomized controlled trials (RCTs) published in English and Chinese from the inception of the databases through August 2024. For the Chinese databases, the search was limited to core journals. Additionally, the references of the included articles were scrutinized to identify any omitted studies. The detailed search strategy is presented in Supplementary Appendix 1.

### 2.3 Eligibility criteria

Inclusion criteria: (1) Study type: randomized controlled trials (RCTs), language limited to English and Chinese. (2) Subjects: college undergraduates, specialists and postgraduates aged 18–28 years old. (3) Interventions: The experimental group received at least 4 weeks of planned physical activity, and the types of exercise included aerobic exercise (including walking, running, cycling, *etc.*), resistance exercise (exercise that overcomes self-weight or external resistance), combined exercise (exercise that alternates aerobic and resistance exercises), high-intensity interval training (HIIT), mind-body exercises (exercises performed by integrating consciousness, breathing and body, such as Baduanjin, yoga, Pilates, etc.), and calisthenics (including all kinds of aerobics, dance, aerobics, etc.).

Exclusion criteria: (1) Literature with duplicated data published. (2) Literature where full text could not be found such as dissertations, review papers, research proposals and conference papers. (3) Acute exercise or animal studies. (4) Exercise mixed with other interventions.

### 2.4 Literature screening and data extraction

Two researchers extracted all data independently, and in case of disagreement, a third researcher intervened to resolve the disagreement by consensus, integrating the opinions of all three. The following information was extracted: lead author, year of publication, age, subject characteristics (number of participants, physical attributes), and intervention details (type of exercise, duration of individual exercise sessions, frequency, and duration of the intervention). In cases of incomplete information or missing data, requests for additional data could be made to the authors via e-mail as necessary.

### 2.5 Risk of bias and GRADE assessment

Two investigators independently assessed the risk of bias (ROB) of the included studies according to the Cochrane Risk of Bias Tool (Higgins et al., 2011). Because blinding participants to an exercise intervention is difficult, this component was not included in the overall ROB score, and a total of six entries were evaluated in this study: (1) allocation generation, (2) concealment of allocation, (3) blinding of outcome assessment, (4) incomplete outcome data addressed, (5) freedom from selective reporting bias, and (6) other forms of bias. The ROB of each study was comprehensively rated on the basis of the risk evaluation of each entry, and the rating methodology was based on existing studies: if there was no high ROB for each of the above entries and the number of unclear ROBs was ≤3, the study was categorized as low ROB; if there was one high ROB, or no high ROB but the number of unclear ROBs was ≥4, the study was categorized as medium ROB; and all other cases were rated as high ROB (Cipriani et al., 2018).

We assessed the certainty of evidence contributing to the network estimates of the outcomes with the Grading of Recommendations Assessment, Development and Evaluation (GRADE) framework.

### 2.6 Methods of counting exercise doses

In this study, dose-response analysis of overall exercise and body composition of different exercise types of interventions was further conducted on the basis of NMA by calculating weekly energy expenditure (i.e., metabolic equivalents, METs) for exercise dosimetry and expressed as METs-min/week. The calculation was performed by first rating the exercise intensity against the exercise level classification scale based on the physiological indices given in the original literature to indicate exercise intensity [maximal heart rate reserve, percentage of maximal heart rate, percentage of maximal oxygen uptake, or perceived exercise intensity (RPE)], and then estimating the METs consumed per minute of exercise in relation to the age and physical characteristics of the subjects (e.g., the presence of underlying diseases, etc.), and finally comparing the METs were multiplied with the single exercise time and the number of exercises per week to obtain METs-min/week (Garber et al., 2011; Ainsworth et al., 2000; Wasfy and Baggish, 2016; Ainsworth et al., 2011). If no exercise intensity indicator was given in the text, METs expended per minute were assessed based on the type of exercise described in the literature against the 2024 Compendium of Physical Activity for Adults (Herrmann et al., 2024), and finally METs-min/week was obtained by multiplying METs with the duration of a single exercise session and the number of exercise sessions per week.

### 2.7 Statistical analysis

#### 2.7.1 Network meta-analysis

In this study, effect sizes were combined using pre- and post-intervention mean and standard deviation change values to minimize the impact of baseline differences. The change in standard deviation was calculated following the Cochrane Handbook (version 6.3) and converted using the formula provided therein. The calculation of combined effect sizes and 95% confidence intervals (CI) was performed using a random-effects model in Stata 17.0 software, adhering to the PRISMA guidelines for network Meta-analysis (Hutton et al., 2015), employing a frequency-based approach (Salanti, 2012). Weighted Mean Difference (WMD) was utilized for calculations, as the units of the primary outcome indicators were consistent. Relationships between movement types were visualized through a network evidence graph, where lines connecting nodes indicate direct comparisons, with line thickness proportional to the number of studies and node size proportional to the sample sizes. The consistency of each closed loop was assessed by calculating the inconsistency factor and its 95% CI (Chaimani et al., 2013). The inconsistency model was applied to test for inconsistency, and if P > 0.05, the consistency model would be employed to continue the analysis (Shim et al., 2017). The efficacy of each intervention type was ranked by the surface under the cumulative ranking (SUCRA), with a SUCRA value of 1 indicating the best effect and 0 indicating the worst effect (Salanti et al., 2011; Mbuagbaw et al., 2017). Funnel plots were inspected for signs of publication bias.

#### 2.7.2 Dose-response analysis

The dose-response relationship analysis between exercise and BMI index was conducted using a random-effects Bayesian model-based Network Meta-Analysis (MBNMA) (Mawdsley et al., 2016). Initially, we validated the key assumptions of MBNMA through network transmissibility (Higgins et al., 2012), data consistency (Wheeler et al., 2010), and network connectivity (Ter et al., 2019). Subsequently, a quadratic model was selected for the dose-response analysis (Shim and Lee, 2019) by comparing the Deviance Information Criterion (DIC) and the fitted plots of each nonlinear model. The standardized mean difference (SMD) was utilized as the effect size measure in the dose analysis, with 95% CI to ascertain validity; results were considered statistically significant if the 95% CI did not encompass 0 (Borg et al., 2024). The dose-response analysis was executed using the “MBNMAdose” package in R (version 4.3.1), and visualization was accomplished using the “ggplot2” package.

## 3 Results

### 3.1 Literature selection

**FIGURE 1:**
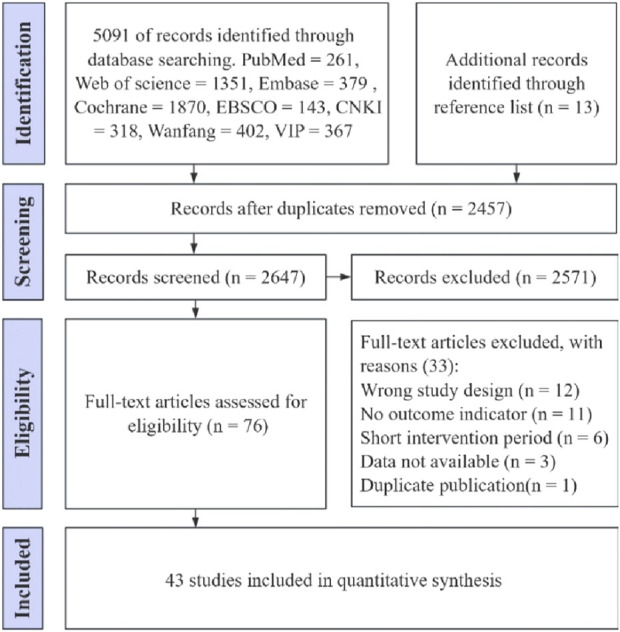
Preferred Reporting Items for Systematic Reviews and Meta-Analyses (PRISMA) flow diagram depicting the study selection process.

As shown in Figure 1, a total of 5,104 potentially eligible studies were identified, with 5,091 coming from searches of eight databases and 13 from reference lists. After removing 2,457 duplicate studies, 2,647 remained for screening. Of these, 2,571 were removed following title and abstract screening, and 33 were removed after downloading and reading the full text, resulting in 43 RCTs included in the analysis (Alexander and Machado, 2024; Chaudhary et al., 2022; Eather et al., 2019; Eimarieskandari et al., 2012; Fisher et al., 2015; Ghorbani et al., 2014; Heydari et al., 2012; Kong et al., 2016; Li et al., 2022; Liu et al., 2023; Moravveji et al., 2019; Nie et al., 2018; Pourabdi et al., 2013; Saltan and Ankaralı, 2021; Sun et al., 2020; Suwannakul et al., 2024; Wang Y. et al., 2023; Xiaolin, 2023; Ye et al., 2022; Zhang et al., 2015; Zhang and Jiang, 2023; An et al., 2022; Cai et al., 2019; Cao et al., 2020; Chen et al., 2020; Chen, 2018; Chen and Zhang, 2022; Fu et al., 2019; Gao et al., 2017; Huang, 2005; Jiao et al., 2021; Li and Chu, 2019; Li et al., 2021; Lin et al., 2016; Liu et al., 2016; Ma and Yuan, 2004; Qi et al., 2013; Xiao et al., 2022; Yan et al., 2011; Yang et al., 2019; Yang and Fu, 2010).

### 3.2 Characteristics of the included studies

The characteristics of the included studies are summarized in Table 1. A total of 3,154 current university students participated in the study, with 2,018 assigned to the experimental group and 1,136 to the control group. The intervention periods across the studies varied from 4 to 40 weeks, with 38 studies (88%) having an intervention period of 8 weeks or longer. The length of a single exercise session spanned from 15 to 90 min, with 36 studies (84%) involving sessions that lasted 30 min or longer. The frequency of exercise sessions per week ranged from 2 to 5, with 38 studies (88%) conducting 3 or more sessions weekly.

**TABLE 1:** Characteristics of the included studies.

Author and published year	Number	Age (mean ± SD)	Subject characteristics	Means of intervention	Exercise duration (minutes)	Period and frequency (week × [times/week])
Alexander 2024	28	20.71 ± 2.76	Sedentary, overweight college students	AE	30	6*3
	10	19.13 ± 1.27		ML		
An 2022	45	19.2 ± 0.7	College girl	CE	50	16*3
	42	19.1 ± 0.8		ML		
Cai 2019	233	20.46 ± 1.52	Overweight college girl	Calisthenics	50	16*3
	246	20.62 ± 1.65		ML		
Cai 2023	15	-	Obese female college students	HIIT	20	8*3
Cai 2023	15	-		AE	60	8*3
Cao 2020	30	-	Obese male college students	AE	45	12*5
Cao 2020	30	-		ML		
Chaudhary 2022	50	-	College student	MBE	60	4*6
Chaudhary 2022	50	-		AE	45	4*6
Chen 2018	23	19.33 ± 1.03	Overweight college girl	Calisthenics	40	16*3
Chen 2018	23	19.16 ± 0.94		ML		
Chen 2019	80	19.81 ± 1.04	College girl	MBE	75	17*2
Chen 2019	80	20.03 ± 1.01		ML		
Chen 2020	20	-	Obese female college students	MBE	40	16*3
Chen 2020	19	-		ML		
Chen 2022	10	-	Overweight college girl	AE	45	10*3
Chen 2022	10	-		HIIT	25	10*3
Chen 2022	7	-		ML		
Eather 2018	27	20.48 ± 2.01	College student	HIIT	10	8*3
Eather 2018	26	20.48 ± 2.01		ML		
Eimarieskandari 2012	7	22.1 ± 0.49	Obese female college students	HIIT	15	8*3
Eimarieskandari 2012	7	22.1 ± 0.49		AE	41	8*3
Eimarieskandari 2012	6	22.1 ± 0.49		ML		
Fisher 2015	15	20.0 ± 1.5	Obese male college students	HIIT	20	6*3
Fisher 2015	13	20.0 ± 1.5		AE	50	6*5
Gao 2017	17	21.6 ± 1.4	Overweight college girl	HIIT	25	12*5
Gao 2017	17	21.6 ± 1.4		AE	40	12*5
Ghorbani 2014	15	26.06 ± 1.18	College girl	AE	40	6
Ghorbani 2014	15	26.33 ± 1.30		ML		
Heydari 2012	25	24.7 ± 4.8	Obese male college students	HIIT	20	12*3
Heydari 2012	21	25.1 ± 3.9		ML		
Huang 2005	12	20.16 ± 1.19	Obese female college students	Calisthenics	60	16*4
Huang 2005	12	20.02 ± 1.21		ML		
Jiao 2021	40	20.0 ± 1.3	College girl	MBE	70	16*5
Jiao 2021	40	20.0 ± 1.3		AE	70	16*5
Kong 2016	11	19.8 ± 0.8	Overweight college girl	HIIT	20	5*4
Kong 2016	11	19.9 ± 2.1		AE	40	5*4
Li 2019	120	20.74 ± 0.71	Overweight college girl	MBE	55	18*3
Li 2019	120	20.32 ± 0.85		ML		
Li 2021	15	20.80 ± 0.86	College girl	MBE	70	8*4
Li 2021	15	20.53 ± 0.64		ML		
Li 2022	14	22.6 ± 2.5	College girl	RE	40	8*2
Li 2022	13	22.5 ± 2.0		ML		
Lin 2016	18	20.1 ± 2.0	Obese female college students	HIIT	20	12*2
Lin 2016	18	20.3 ± 2.2		ML		
Liu 2016	20	-	Obese female college students	HIIT	15	12*4
Liu 2016	20	-		AE	30	12*4
Liu 2023	20	20.09 ± 1.35	Obese female college students	AE	-	8
Liu 2023	20	20.08 ± 1.76		ML		
Ma 2004	30	18.7 ± 0.65	College girl	MBE	60	16*3
Ma 2004	30	18.97 ± 0.89		ML		
Moravveji 2019	12	20.33 ± 1.49	Overweight college girl	AE	45	8*3
Moravveji 2019	12	20.66 ± 2.24		ML		
Nie 2018	17	21.0 ± 1.1	Overweight college girl	HIIT	-	8*3
Nie 2018	15	20.9 ± 1.6		AE	-	8*3
Nie 2018	16	20.8 ± 1.1		ML		
Pour-Abdi 2013	16	-	Overweight college girl	HIIT	28	6*3
Pour-Abdi 2013	10	-		ML		
Qi 2013	20	-	Obese female college students	HIIT	25	12*5
Qi 2013	20	-		AE	50	12*5
Qi 2013	20	-		ML		
Saltan 2020	35	18.82 ± 1.071	College student	MBE	45	12*3
Saltan 2020	35	18.85 ± 2.495		CE	45	12*3
Saltan 2020	35	19.42 ± 1.378		ML		
Sun 2020	150	21.78 ± 1.47	Obese male college students	HIIT	30	12*5
Sun 2020	150	21.63 ± 1.39		AE	40	12*5
Suwannakul 2024	22	20.29 ± 0.96	Obese female college students	MBE	40	8*3
Suwannakul 2024	22	20.36 ± 1.05		ML		
wang 2023	30	19.91 ± 1.57	College student	HIIT	30	8*3
wang 2023	30	19.59 ± 1.53		CE	30	8*3
Xiao 2022	22	-	Obese college students	AE	60	10*4
Xiao 2022	22	-		CE		
Xiao 2022	22	-		ML		
Xiong 2011	40	19.94 ± 1.12	College girl	MBE	90	12*3
Xiong 2011	40	20.12 ± 1.05		ML		
Yang 2010	30	18.2 ± 2.3	Obese female college students	Calisthenics	60	40*4
Yang 2010	30	18.2 ± 2.3		ML		
Yang 2019	18	-	Obese college students	AE	60	12*3
Yang 2019	19	-		RE	60	12*3
Yang 2019	20	-		CE	60	12*3
Ye 2022	65	18.90 ± 1.02)	College student	MBE	50	12*4
Ye 2022	65	18.80 ± 0.99		ML		
Zhang 2009	69	19.63 ± 1.45	College girl	Calisthenics	60	16*3
Zhang 2009	77	19.58 ± 1.52		ML		
Zhang 2015	14	21.0 ± 1.0	Overweight college girl	HIIT	25	12*4
Zhang 2015	15	20.6 ± 1.2		AE	33	12*4
Zhang 2015	14	20.9 ± 1.0		ML		
Zhang 2023	39	19.23 ± 0.98	College student	MBE	40	12*3
Zhang 2023	39	19.16 ± 1.05		ML		
Zhao 2019	12	21.68 ± 0.98	Obese male college students	Calisthenics	50	12*5
Zhao 2019	12	21.42 ± 1.14		CE	50	12*5
Zhao 2019	12	21.45 ± 1.02		ML		

ML: maintain lifestyle; AE: aerobic exercise; RE: resistance exercise; MBE: Mind-body exercise; HIIT: high-intensity interval training.

### 3.3 Results of risk of bias assessment

The bias risk assessment results are detailed in Supplementary Appendix 2. Among the studies, 42 detailed their allocation methods, 21 described their allocation concealment, and 10 reported blinding of outcome assessments. Dropout rates were reported by 34 studies; one of these, with a dropout rate exceeding 20%, was rated as high risk. The remaining 40 studies indicated a low risk of selective reporting. In terms of other biases, four studies were rated high risk due to small sample sizes (fewer than 10 participants in any group). Overall, the studies were rated as follows: 37 low risk, 4 medium risk, and 2 high risk.

### 3.4 Results of network meta-analysis

**FIGURE 2:**
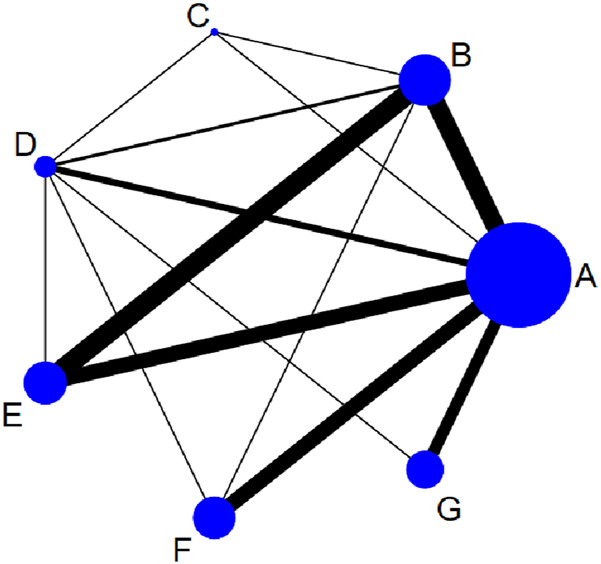
Network plot presenting the effects of different exercise types on body composition in university students. **(A)**: Control; **(B)**: Aerobic exercise; **(C)**: Resistance exercise; **(D)**: Combined exercise; **(E)**: HIIT; **(F)**: Mind-body exercise; **(G)**: Calisthenics.

Figure 2 displays the NMA plots for eligible studies examining the effects of various exercise types on body composition. As shown in the plots, aerobic exercise was the most frequently included intervention, whereas resistance exercise was the least common.

Supplementary Appendix 3 illustrates the contributions of direct and indirect comparisons to the NMA, as well as the number of studies involved in each direct comparison. All outcome indicators were assessed using loop-specific heterogeneity estimates, an inconsistency model, and node-splitting analysis, as detailed in Supplementary Appendix 4.

The loop-specific heterogeneity estimates demonstrated good consistency within each closed loop for depression, anxiety, and stress. The inconsistency model showed p-values greater than 0.05 for all three conditions, suggesting no significant inconsistency. The node-splitting analysis also revealed no inconsistencies in either direct or indirect evidence, confirming the reliability of the results.

The results of the network meta-analysis are presented in Table 2. Compared with the control group, aerobic exercise [MD = −1.04, 95% CI (−1.48, −0.61), P < 0.0001], combined exercise [MD = −1.31, 95% CI (−1.92, −0.69), P < 0.0001], HIIT [MD = −1.03, 95% CI (−1.50, −0.55), P < 0.0001], physical and mental exercise [MD = −1.28, 95% CI (−1.81, −0.74), P < 0.0001], and calisthenics [MD = −1.50, 95% CI (−2.08, −0.91), P < 0.0001] had a significant effect on college students’ body composition. In cross-comparisons, no significant differences were found between any two exercise types. Supplementary Appendix 5 displays a forest plot of the outcome metrics, including 95% CI and 95% prediction intervals (95% PI).

**TABLE 2:** Results of network Meta-analysis.

B						
−0.50 (−1.59.0.59)	C					
0.26 (−0.40.0.93)	0.76 (−0.35.1.88)	D				
−0.02 (−0.45.0.42)	0.48 (−0.64.1.61)	−0.28 (−0.97.0.42)	E			
0.23 (−0.43.0.90)	0.73 (−0.46.1.93)	−0.03 (−0.82.0.76)	0.25 (−0.45.0.95)	F		
0.45 (−0.27.1.17)	0.95 (−0.26.2.16)	0.19 (−0.62.0.99)	0.47 (−0.28.1.21)	0.22 (−0.57.1.01)	G	
−1.04 (-1.48,-0.61)	−0.54 (−1.62.0.53)	−1.31 (-1.92,-0.69)	−1.03 (-1.50,-0.55)	−1.28 (-1.81,-0.74)	−1.50 (-2.08,-0.91)	A

A: control; B: aerobic exercise; C: resistance exercise; D: combined exercise; E: HIIT; F: Mind-body exercise; G: calisthenics.

**FIGURE 3:**
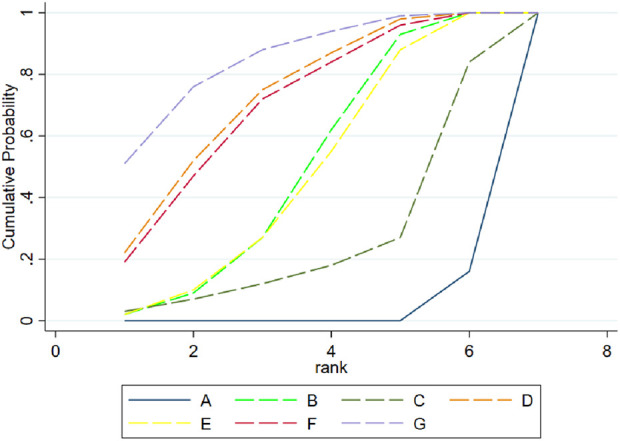
SUCRA Probability Ranking Chart, the figure shows the ranking of the effects of each exercise type on the body composition interventions. **(A)** Control; **(B)** Aerobic exercise; **(C)** Resistance exercise; **(D)** Combined exercise; **(E)** HIIT; **(F)** Mind-body exercise; **(G)** Calisthenics.

The SUCRA probability ranking is depicted in Figure 3, with the detailed SUCRA ranking results provided in Supplementary Appendix 3. The exercise type with the highest probability of being the most effective for reducing BMI was calisthenics, with a SUCRA value of 84.7%. This was followed by combined exercise, with a SUCRA value of 72.4%. Conversely, resistance exercise had the lowest probability of being effective in influencing body composition, with a SUCRA value of 24.9%.

**FIGURE 4:**
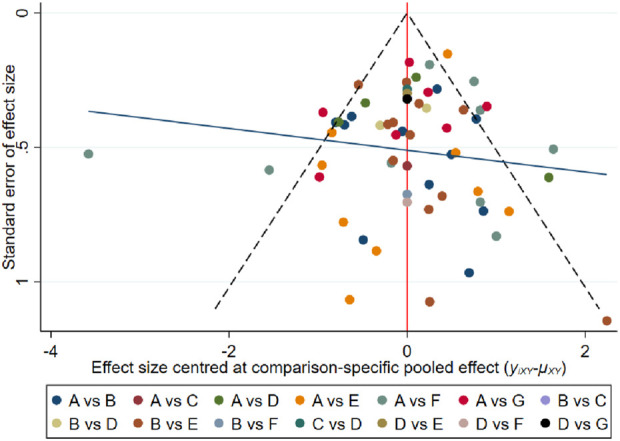
Funnel plot of network meta-analysis, according to this figure, the publication bias status of the included literature can be analyzed. **(A)** Control; **(B)** Aerobic exercise; **(C)** Resistance exercise; **(D)** Combined exercise; **(E)** HIIT; **(F)** Mind-body exercise; **(G)** Calisthenics.

The analysis funnel plot (Figure 4) was tested for publication bias for the outcome indicators, and it was found that the symmetry of the funnel plot for the outcome indicators was better, with less impact of publication bias or small sample effect.

### 3.5 Dose-response results

The key assumptions for network meta-analysis were first validated through assessments of network connectivity, data consistency, and transferability (Ter et al., 2019; Donegan et al., 2013; Watt et al., 2019), as detailed in Supplementary Appendix 7. Subsequently, by comparing the Deviance Information Criterion (DIC) and fitted plots of various models—including Emax, restricted cubic spline, and nonparametric models—we selected the quadratic model as the analytical model for this study (Supplementary Appendix 8). Furthermore, we conducted a series of tests to ensure the stability of the chosen model, as described in Supplementary Appendix 8 (Mawdsley et al., 2016; Dias et al., 2013).

**FIGURE 5:**
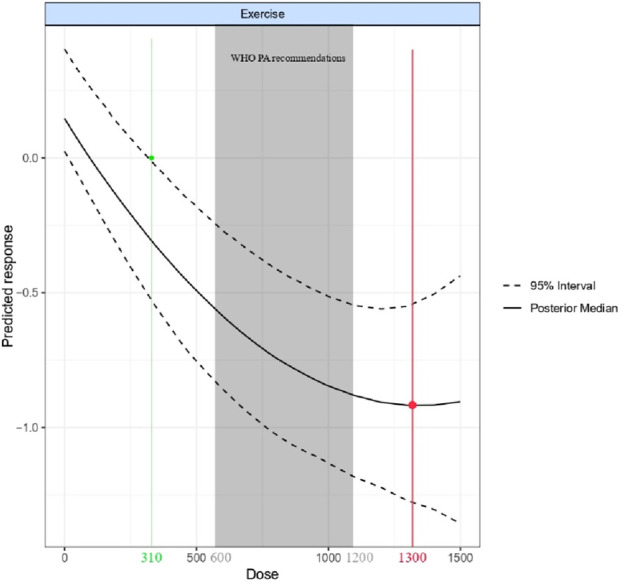
Overall exercise dose-response relationship. The green line is the minimum response measure that produces a significant effect. The red line is the dose that produces the maximum response effect. The shaded area is the WHO recommended range of physical activity.

Figure 5 illustrates the nonlinear dose-response relationship between overall exercise and body composition. The predictions indicate a significant response to the effect of holistic exercise on body composition starting at 310 METs-min/week (where the 95% CI upper limit is less than 0), with the maximum effect achieved when the exercise dose reached 1,300 METs-min/week (SMD = −0.91; 95% CI [-1.25, −0.54]; SD = 0.19). The effect of exercise on body composition exhibited a moderate effect at doses up to 600 METs-min/week [equivalent to the energy expenditure at the lower limit of physical activity recommended by the World Health Organization (WHO); SMD = −0.59; 95% CI (−0.87, −0.28); SD = 0.15], and a strong effect at doses up to 1,200 METs-min/week [equivalent to the upper limit of physical activity recommended by the WHO; SMD = −0.9; 95% CI (−1.22, −0.55); SD = 0.17] (Bull et al., 2020).

**FIGURE 6:**
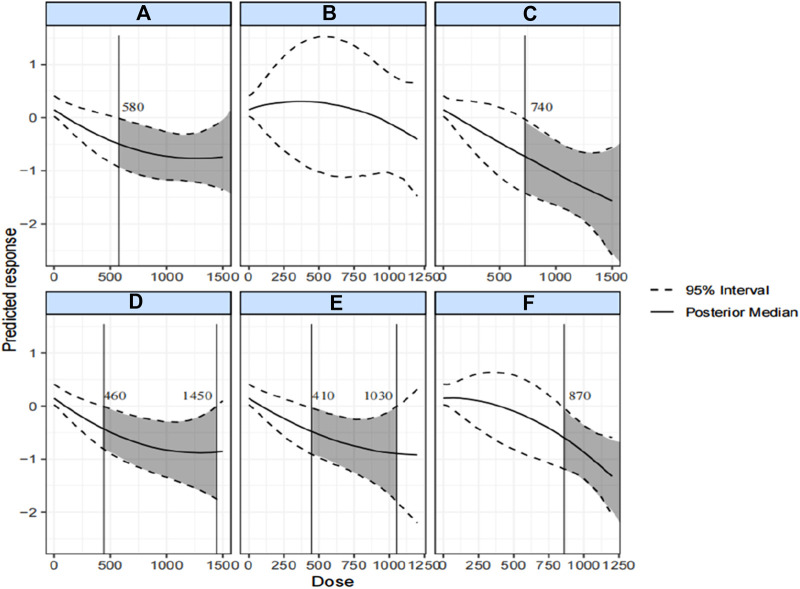
Dose-response relationship by exercise type. The black line is the minimum response measure that produces a significant effect. The shaded area is the WHO recommended range of physical activity. **(A)** Aerobic exercise; **(B)** Resistance exercise; **(C)** Combined exercise; **(D)** HIIT; **(E)** Mind-body exercise; **(F)** Calisthenics.

Figure 6 illustrates the dose-response relationships for six types of exercise affecting body composition. The predicted results indicated a statistically non-significant dose-response relationship only for resistance exercise. In contrast, aerobic, combined, HIIT, mind-body, and calisthenics exercises showed non-linear dose-response relationships.

Mind-body exercise had the lowest minimum significant dose at 410 METs-min/week (SMD = −0.44), but the significant effect ceased at doses up to 1,030 METs-min/week (SMD = −0.88). Aerobic exercise required the highest minimum significant dose of 870 METs-min/week (SMD = −0.61), with its effect on body composition increasing more rapidly as the exercise dose increased. The minimum significant dose for aerobic exercise was 580 METs-min/week (SMD = −0.49), and its effect increased very gradually as the exercise dose exceeded 1,300 METs-min/week (SMD = −0.76). Combined exercise had a minimum response dose of 740 METs-min/week (SMD = −0.76), with its effect increasing more smoothly and gradually with increasing exercise dose. For HIIT, the minimum response dose was 460 METs-min/week (SMD = −0.44), and the significant effect disappeared when the dose was increased to 1,450 METs-min/week (SMD = −0.87). A plot of the dose-response relationship, including the original study dataset, is provided in Supplementary Appendix 9.

The ranked results in Supplementary Appendix 10 show that combined exercise (1,500 METs-min/week) had the highest probability of producing the greatest impact (SMD = −1.56), the second exercise was calisthenics (1,200 METs-min/week; SMD = −1.32), and the last ranked was resistance exercise.

### 3.6 GRADE assessment

Supplementary Appendix 11 shows the SUCRA-ranked GRADE assessments for each comparison for each outcome indicator and for the treatment measures for the outcome indicators. Overall, most of the comparative GRADE assessment ratings were rated as “Low.” SUCRA GRADE assessments were rated as “Moderate”.

## 4 Discussion

This systematic review and NMA encompassed 43 RCTs involving 3,154 participants, aiming to evaluate the impact of various exercise types on body composition in college students. Aerobic exercise, combined exercise, HIIT, mind-body exercise, and calisthenics all significantly reduced BMI. However, no significant differences in effectiveness were observed between the types of exercise. Additionally, dose-response analyses predicted changes in the effects of overall exercise and each specific type on body composition, revealing a nonlinear relationship between exercise dose and body composition. The analyses also indicated a low dose threshold for the impact of exercise interventions on body composition.

### 4.1 Results of network meta-analysis

After entering college, lifestyle changes and increased sedentary behavior have contributed to rising obesity rates among college students (Lv et al., 2019; Vankim and Nelson, 2013). Although numerous studies have shown that different types of exercise have varying effects on body composition, the results are inconsistent (Irandoust et al., 2022). It is still unclear which type of exercise is most effective for altering body composition in college students and what exercise dose is most beneficial when designing a training program. By incorporating a large number of RCTs and conducting both direct and indirect analyses, this study determined that aerobic exercise, combined exercise, HIIT, mind-body exercise, and calisthenics all significantly reduced BMI in college students. The SUCRA results indicate the following ranking: Calisthenics > Combined exercise > Mind-body exercise > Aerobic exercise > HIIT > Resistance exercise. Calisthenics had the highest likelihood of being the most effective exercise type, while resistance exercise was most likely to be the least effective for this population. These findings align with the analyses by Morze et al. and Wang et al. (Morze et al., 2021; Wang et al., 2022), although their studies included all adults. Also our study is in line with the findings of AL-Mhanna et al. (Al-Mhanna et al., 2024a; Al-Mhanna et al., 2024b) and Batrakoulis et al. (Batrakoulis et al., 2022) that Combined exercise is the type of exercise that has a significant effect in intervening in obesity.

### 4.2 Results of dose-response analysis

We also discovered a nonlinear dose-response relationship between overall exercise and BMI for all types of exercise. The minimum effective dose for overall exercise was estimated to be 310 METs-min/week, a threshold that is quite low and equivalent to 90 min of slow walking (3.5 METs-min) or 80 min of cycling (4 METs-min). The maximum response dose is anticipated to be 1,300 METs-min/week, which is equivalent to 170 min of jogging (7.5 METs-min) or 160 min of basketball or badminton (8 METs-min) (Herrmann et al., 2024). College students who engage in these activities for an average of 20–25 min per day outside of school hours can effectively improve their body composition and lower their BMI. Beyond 1,300 METs-min/week, the increase in the intervention effect becomes very gradual. Our findings suggest that exceeding the physical activity doses recommended by the WHO can provide additional benefits in reducing the BMI index among college students (Bull et al., 2020). Therefore, college students should aim to exercise up to 1,300 METs-min/week of energy expenditure, as time and physical condition permit.

In addition, we observed varying dose-response relationships between different exercise types and BMI. The dose-response relationships for aerobic exercise, combined exercise, HIIT, physical and mental exercise, and calisthenics were all statistically significant. The intervention effects of aerobic exercise, combined exercise, and calisthenics progressively strengthened with increasing exercise dose. However, the effects of HIIT and physical and mental exercise lost significance after reaching certain doses of 1,450 METs-min/week and 1,030 METs-min/week, respectively. Ranking analyses revealed that combined exercise (at 1,500 METs-min/week) had the highest probability of producing the greatest effect (SMD = −1.56), followed by calisthenics (at 1,200 METs-min/week; SMD = −1.32). Resistance exercise was the last in the ranking. These findings align with the SUCRA probability ranking results.

### 4.3 Strengths and limitations

The strengths of this study are threefold. Firstly, we synthesized both direct and indirect comparisons to rank the effectiveness of various exercise types on college students’ body composition, offering new and more reliable evidence. Secondly, our study recognized that both exercise type and dose are crucial factors in exercise program development. By integrating network meta-analysis with a novel dose-response network analysis, we examined the dose-response relationships between overall exercise, individual exercise types, and BMI. This combination of methods enabled us to more comprehensively predict the optimal exercise prescription for lowering BMI in college students. Lastly, all included studies were RCTs, enhancing the reliability of our evidence.

However, the study is not without limitations. Firstly, of the included RCTs, only 10 reported the use of blinding in outcome assessment, with 2 rated as high risk of bias and 4 as medium risk. This could have introduced some degree of error. Secondly, the limitation of this study also lies in the fact that subgroup analyses were not conducted based on factors such as gender, geographic location, and duration and frequency of exercise interventions, and this aspect was focused on in subsequent studies to enhance the applicability of the findings to different populations. Again, the population selected for this study was a group of college students, and the findings are less applicable to other age groups; in subsequent studies, attention should be paid to conducting research based on subjects of different ages. Finally, this study only examined the effect of a single intervention, exercise, on body composition, and future studies will have to draw on Al Mhanna et al.'s (Al-Mhanna et al., 2023) study to explore the optimal intervention for intervening on body composition in conjunction with diet.

## Data Availability

The original contributions presented in the study are included in the article/supplementary material, further inquiries can be directed to the corresponding authors.
