# Supplementary material for: Comparative efficacy of different exercise types on body composition in university students: a systematic review and meta-analysis of randomized controlled trials
Source: Front Physiol. 2025 Apr 22;16:1537937. doi: 10.3389/fphys.2025.1537937 (PMC12053280; doi:10.3389/fphys.2025.1537937)
Supplement: Supplementary file 1 [file Table1.docx]

**Comparative Efficacy of Different Exercise Types on Body Composition in University Students: A Systematic Review and Meta-Analysis of Randomized Controlled Trials**

**Jihai Li, Liuhong Zang, Sihai Hao, Hui Wang***

Institute of Physical Education, Xinjiang Normal University, Urumqi, Xinjiang, 830054, China

*Corresponding author. Hui Wang, Institute of Physical Education, Xinjiang Normal University, Urumqi 830054, China.

Email address: 1006363016@qq.com

**Appendix 1.** Search strategy.

| **Database** | **Search condition** | | | |
| --- | --- | --- | --- | --- |
|  | **Exercise** | **University student** | **Body composition** | **Randomized controlled trials** |
| PubMed [Title/Abstract] | Exercise [MeSH Terms] OR exerci* OR sport* OR aerobic* OR physical exerci* OR physical activi* OR physical train* OR circuit training OR physical endurance OR physical fitness OR resistance training OR strength training | Students [MeSH Terms] OR student* AND Universities [MeSH Terms] OR undergrad* OR college* OR education OR tertiary* OR universit* | Obesity [MeSH Terms] OR overweight OR BMI OR body mass index OR body weight OR fat mass | randomized controlled trial OR randomized OR placebo OR RCT |
| Cochrane [Title/Abstract/ keywords] | Exercise [MeSH Terms] OR exerci* OR sport* OR aerobic* OR physical exerci* OR physical activi* OR physical train* OR circuit training OR physical endurance OR physical fitness OR resistance training OR strength training | Students [MeSH Terms] OR student* AND Universities [MeSH Terms] OR undergrad* OR college* OR education OR tertiary* OR universit* | Obesity [MeSH Terms] OR overweight OR BMI OR body mass index OR body weight OR fat mass | randomized controlled trial OR randomized OR placebo OR RCT |
| Embase [Title/Abstract] | Exercise [Emtree term] OR exerci* OR sport* OR aerobic* OR physical exerci* OR physical activi* OR physical train* OR circuit training OR physical endurance OR physical fitness OR resistance training OR strength training | Students [Emtree term] OR student* AND Universities [Emtree term] OR undergrad* OR college* OR education OR tertiary* OR universit* | Obesity [Emtree term] OR overweight OR BMI OR body mass index OR body weight OR fat mass | randomized controlled trial OR randomized OR placebo OR RCT |
| Web of Science [Topic] | exercise OR exerci* OR sport* OR aerobic* OR physical exerci* OR physical activi* OR physical train* OR circuit training OR physical endurance OR physical fitness OR resistance training OR strength training | students OR student* AND universities OR undergrad* OR college* OR education OR tertiary* OR universit* | Obesity OR overweight OR BMI OR body mass index OR body weight OR fat mass | randomized controlled trial OR randomized OR placebo OR RCT |
| EBSCO [Abstract] | exercise OR exerci* OR sport* OR aerobic* OR physical exerci* OR physical activi* OR physical train* OR circuit training OR physical endurance OR physical fitness OR resistance training OR strength training | students OR student* AND universities OR undergrad* OR college* OR education OR tertiary* OR universit* | Obesity OR overweight OR BMI OR body mass index OR body weight OR fat mass | randomized controlled trial OR randomized OR placebo OR RCT |
| CNKI [Title/Abstract/ keywords] | exercise OR sport OR circuit training OR physical endurance OR physical fitness OR resistance training OR strength training | students AND universities OR education | Obesity OR overweight OR BMI OR body mass index OR body weight OR fat mass | randomized controlled trial OR randomized OR placebo OR RCT |
| Wanfang [Title/Abstract/ keywords] | exercise OR sport OR circuit training OR physical endurance OR physical fitness OR resistance training OR strength training | students AND universities OR education | Obesity OR overweight OR BMI OR body mass index OR body weight OR fat mass | randomized controlled trial OR randomized OR placebo OR RCT |
| VIP [Title/Abstract/ keywords] | exercise OR sport OR circuit training OR physical endurance OR physical fitness OR resistance training OR strength training | students AND universities OR education | Obesity OR overweight OR BMI OR body mass index OR body weight OR fat mass | randomized controlled trial OR randomized OR placebo OR RCT |

**Appendix 2.** Risk of bias assessment.

| **Study** | **Allocation generation** | **Concealment of allocation** | **Blinding of outcome assessment** | **Dropout rate %** | **Selective reporting** | **Other bias** | **Risk category** |
| --- | --- | --- | --- | --- | --- | --- | --- |
| Alexander 2024 | Low | Low | Unclear | 16% | Low | Low | Low |
| An 2022 | Low | Unclear | Unclear | 0% | Low | Low | Low |
| Cai 2019 | Low | Unclear | Unclear | 0% | Low | Low | Low |
| Cai 2023 | Low | Unclear | Unclear | 0% | Low | Unclear | Low |
| Cao 2020 | Low | Unclear | Unclear | Unclear | Low | Low | Low |
| Chaudhary 2022 | Low | Unclear | Unclear | Unclear | Low | Unclear | Moderate |
| Chen 2018 | Low | Unclear | Unclear | Unclear | Low | Low | Low |
| Chen 2019 | Low | Low | Unclear | Unclear | Low | Low | Low |
| Chen 2020 | Low | Unclear | Unclear | 0% | Low | Low | Low |
| Chen 2022 | Low | Low | Unclear | 0% | Low | High | Moderate |
| Eather 2018 | Low | Low | Low | 0% | Low | Low | Low |
| Eimarieskandari 2012 | Low | Unclear | Unclear | 0% | Low | High | Moderate |
| Fisher 2015 | Low | Low | Unclear | 18% | Low | Low | Low |
| Gao 2017 | Low | Unclear | Unclear | 0% | Low | Low | Low |
| Ghorbani 2014 | Low | Low | Unclear | Unclear | Low | Low | Low |
| Heydari 2012 | Low | Unclear | Unclear | 17% | Low | Low | Low |
| Huang 2005 | Low | Unclear | Unclear | 0% | Low | Low | Low |
| Jiao 2021 | Low | Unclear | Unclear | 0% | Low | Unclear | Low |
| Kong 2016 | Low | Unclear | Unclear | 14% | Low | High | Low |
| Li 2019 | Low | Low | Unclear | 0% | Low | Low | Low |
| Li 2021 | Low | Low | Unclear | 0% | Unclear | Low | Low |
| Li 2022 | Low | Low | Low | 0% | Low | Low | Low |
| Lin 2016 | Low | Low | Unclear | 0% | Low | Low | Low |
| Liu 2016 | Low | Unclear | Unclear | 0% | Unclear | Low | Low |
| Liu 2023 | Low | Low | Unclear | 5% | Low | Low | Low |
| Ma 2004 | Low | Low | Unclear | Unclear | Low | Low | Low |
| Moravveji 2019 | Low | Low | Low | 21% | Low | High | High |
| Nie 2018 | Low | Unclear | Unclear | 10% | Low | Low | Low |
| Pour-Abdi 2013 | Low | Unclear | Unclear | Unclear | Low | Low | Low |
| Qi 2013 | Low | Unclear | Unclear | 13% | Low | Low | Low |
| Saltan 2020 | Low | Low | Low | 12% | Low | Low | Low |
| Sun 2020 | Low | Low | Unclear | 0% | Unclear | Low | Low |
| Suwannakul 2024 | Low | Low | Low | 3% | Low | Low | Low |
| wang 2023 | Low | Low | Low | 0% | Low | Low | Low |
| Xiao 2022 | Low | Unclear | Unclear | 0% | Low | Unclear | Low |
| Xiong 2011 | Low | Low | Low | 0% | Low | Low | Low |
| Yang 2010 | Low | Unclear | Unclear | Unclear | Low | Unclear | Moderate |
| Yang 2019 | Low | Low | Unclear | 0% | Low | Low | Low |
| Ye 2022 | Low | Low | Low | 8% | Low | Low | Low |
| Zhang 2009 | Unclear | Unclear | Unclear | Unclear | Low | Unclear | High |
| Zhang 2015 | Low | Unclear | Low | 19% | Low | Low | Low |
| Zhang 2023 | Low | Low | Low | 8% | Low | Low | Low |
| Zhao 2019 | Low | Unclear | Unclear | 0% | Low | Low | Low |

**Appendix 3.** Contributions of direct and indirect comparisons to NMA and the number of studies of each direct comparison.


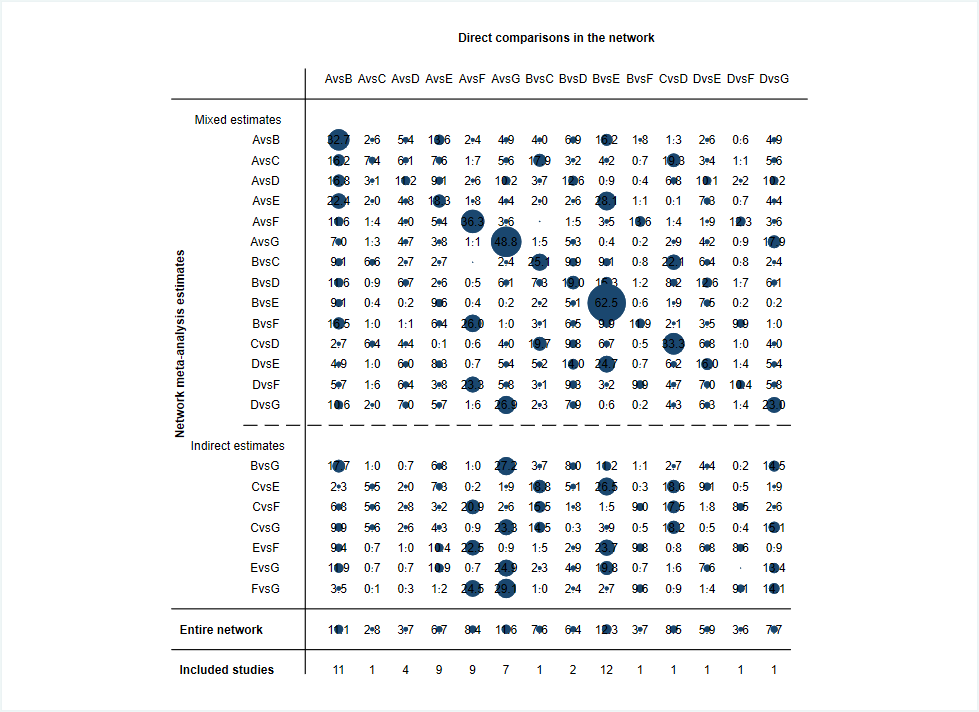


A: Control; B: Aerobic exercise; C: Resistance exercise; D: Combined exercise; E: HIIT; F: Mind-body exercise; G: Calisthenics.

**Appendix 4.** Inconsistency of outcome indicator tested by loop-specific heterogeneity estimates, inconsistency model and node splitting analysis.

**Note:** A: Control; B: Aerobic exercise; C: Resistance exercise; D: Combined exercise; E: HIIT; F: Mind-body exercise; G: Calisthenics..

| **loop-specific heterogeneity estimates** | | | | | | |
| --- | --- | --- | --- | --- | --- | --- |
| **Loop** | **IF** | **seIF** | **z_value** | **p_value** | **CI_95** | **Loop_Heterog_tau2** |
| A-C-D | 1.251 | 1.461 | 0.856 | 0.392 | (0.00,4.12) | 0.404 |
| A-B-C | 0.877 | 0.897 | 0.977 | 0.329 | (0.00,2.64) | 0.172 |
| B-D-F | 0.833 | 1.012 | 0.823 | 0.41 | (0.00,2.82) | 0 |
| A-B-F | 0.602 | 1.366 | 0.441 | 0.66 | (0.00,3.28) | 0.808 |
| B-C-D | 0.52 | 0.595 | 0.873 | 0.383 | (0.00,1.69) | 0 |
| A-D-E | 0.311 | 0.837 | 0.372 | 0.71 | (0.00,1.95) | 0.34 |
| A-D-F | 0.305 | 1.782 | 0.171 | 0.864 | (0.00,3.80) | 1.048 |
| A-B-D | 0.253 | 0.588 | 0.431 | 0.667 | (0.00,1.41) | 0.219 |
| A-D-G | 0.184 | 0.925 | 0.199 | 0.842 | (0.00,2.00) | 0.337 |
| A-B-E | 0.08 | 0.382 | 0.21 | 0.833 | (0.00,0.83) | 0.184 |
| B-D-E | 0.056 | 0.452 | 0.124 | 0.901 | (0.00,0.94) | 0.017 |

**Appendix 4-1**

| **Inconsistency model** | |
| --- | --- |
| **chi2** | **Prob > chi2** |
| **11.2** | **0.262** |

**Appendix 4-2**

| **Node splitting analysis** | | | | | | | |
| --- | --- | --- | --- | --- | --- | --- | --- |
| **Side** | **Direct** | | **Indirect** | | **Difference** | |  |
|  | **Coef.** | **Std. Err.** | **Coef.** | **Std. Err.** | **Coef.** | **Std. Err.** | **P>\|z\|** |
| A B | -1.08397 | 0.267215 | -0.95522 | 0.399741 | -0.12874 | 0.480527 | 0.789 |
| A C | 0.1 | 0.904377 | -0.91792 | 0.690379 | 1.017921 | 1.13777 | 0.371 |
| A D | -1.36541 | 0.40806 | -1.21907 | 0.496183 | -0.14633 | 0.63522 | 0.818 |
| A E | -1.11597 | 0.312223 | -0.88096 | 0.401456 | -0.23501 | 0.508687 | 0.644 |
| A F | -1.19509 | 0.285191 | -2.10321 | 0.883562 | 0.908127 | 0.925475 | 0.326 |
| A G * | -1.50884 | 0.30761 | -1.16736 | 1.513757 | -0.34147 | 1.540167 | 0.825 |
| B C | 0.306214 | 0.779435 | 0.722587 | 0.824657 | -0.41637 | 1.144647 | 0.716 |
| B D | -0.18026 | 0.574106 | -0.30904 | 0.432162 | 0.128778 | 0.718405 | 0.858 |
| B E | 0.101042 | 0.253415 | -0.28669 | 0.479374 | 0.387735 | 0.5413 | 0.474 |
| B F | -0.74002 | 0.977077 | -0.16564 | 0.362309 | -0.57437 | 1.042088 | 0.582 |
| C D | -0.26655 | 0.756012 | -1.41664 | 0.867592 | 1.150088 | 1.156272 | 0.32 |
| D E | 0.059995 | 0.774735 | 0.340026 | 0.40643 | -0.28003 | 0.874872 | 0.749 |
| D F | 0.414215 | 0.996651 | -0.04904 | 0.444733 | 0.463256 | 1.089323 | 0.671 |
| D G | 0.066115 | 0.782397 | -0.29057 | 0.492251 | 0.356688 | 0.92469 | 0.7 |

**Appendix 4-3**

**Appendix 5.** Forest plots of eligible comparisons of outcome indicator.


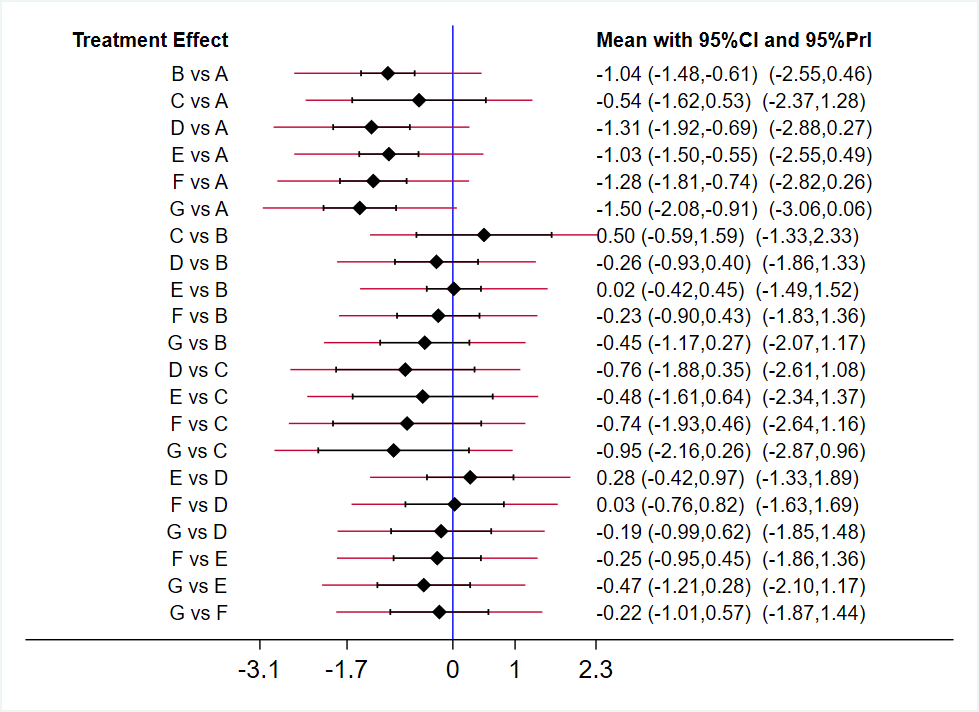


A: Control; B: Aerobic exercise; C: Resistance exercise; D: Combined exercise; E: HIIT; F: Mind-body exercise; G: Calisthenics.

**Appendix 6** SUCRA probability ranking results

| **Type of exercise** | B | C | D | E | F | G |
| --- | --- | --- | --- | --- | --- | --- |
| SUCRA | 48.6 | 24.9 | 72.4 | 47 | 69.7 | 84.7 |

A: Control; B: Aerobic exercise; C: Resistance exercise; D: Combined exercise; E: HIIT; F: Mind-body exercise; G: Calisthenics.

**Appendix 7.** Key assumptions of Network Meta-Analysis

There are three key assumptions to conduct a Network Meta-Analysis (NMA): (1) network connectivity, (2) consistency in the data, and (3) transitivity(Donegan et al., 2013; Watt et al., 2019).

**1.Connectivity**

Connectivity is a key assumption in NMA which if deemed insufficient (i.e., due to lack of direct comparators) can lead to low statistical power and misleading results(Ter Veer et al., 2019). In our study, we assessed connectivity of the network at both treatment and agent levels visually and found no evidence of unconnectedness on either network (Appendix 7-1 and Appendix 7-2).

**Appendix 7-1** Treatment-level network.


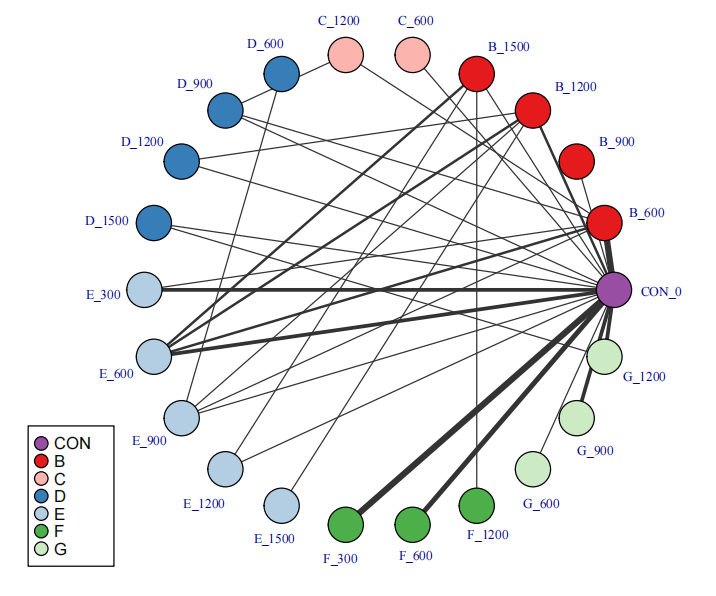


The numbers indicate the specific dose of that intervention. CON: Control; B: Aerobic exercise; C: Resistance exercise; D: Combined exercise; E: HIIT; F: Mind-body exercise; G: Calisthenics.


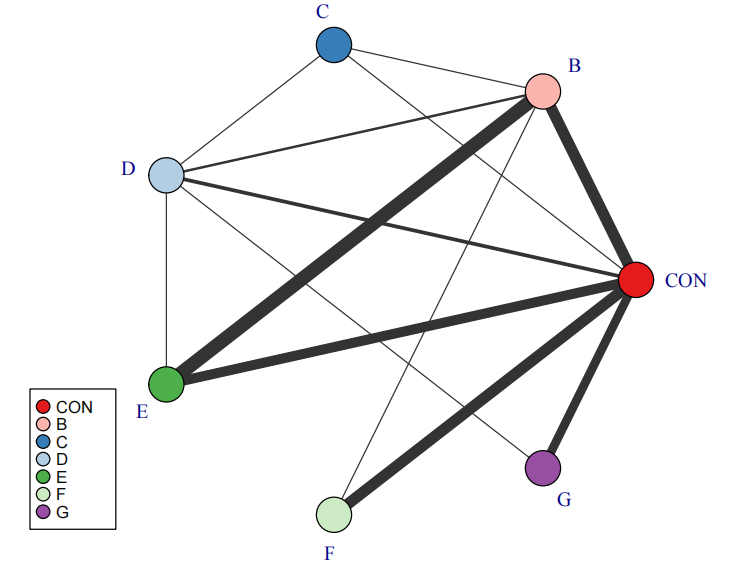


The first value indicates the specific intervention and the second one is the corresponding dose of that intervention (METs-min per week).

CON: Control; B: Aerobic exercise; C: Resistance exercise; D: Combined exercise; E: HIIT; F: Mind-body exercise; G: Calisthenics.

**2.Consistency**

We carried out consistency analysis in the data through the comparison of consistent (i.e., network effect sizes) and unrelated mean effects (UME) models (i.e., pairwise effect sizes) of the network.

In practice, we checked whether deviance, the number of estimated parameters in the network, and the Deviance Informative Criterion (DIC) indicators were similar for both models which would indicate a good fit(Wheeler et al., 2010). Comparison of these parameters indicated good consistency across models (**Appendix 7-3**).

**Appendix 7-3** Consistent and UME models fit comparison

| **Model** | **pD** | **Residual deviance** | **DIC** | **SD** |
| --- | --- | --- | --- | --- |
| Consistent | 82.2 | 88.34 | 122.1 | 0.55 |
| UME | 78.5 | 87.735 | 117.8 | 0.51 |

pD: Number of estimated parameters; DIC: Deviance Informative Criterion; SD: Standard Deviation; UME: Unrelated Mean Effects. Scientific literature indicated that the main indicator to assess the model fit is the DIC. As lower DIC, better fit.

**3.Transitivity**

We assessed transitivity *via* MBNMA node-splitting approach. This method splits and compares contributions for a particular treatment contrast into direct and indirect evidence(van Valkenhoef et al., 2016). Similar effects denote good transitivity. Appendix 7-4 (density plots) below present the results for transitivity in this meta-analysis.

**Appendix 7-4** Node-splitting analysis (density plot).


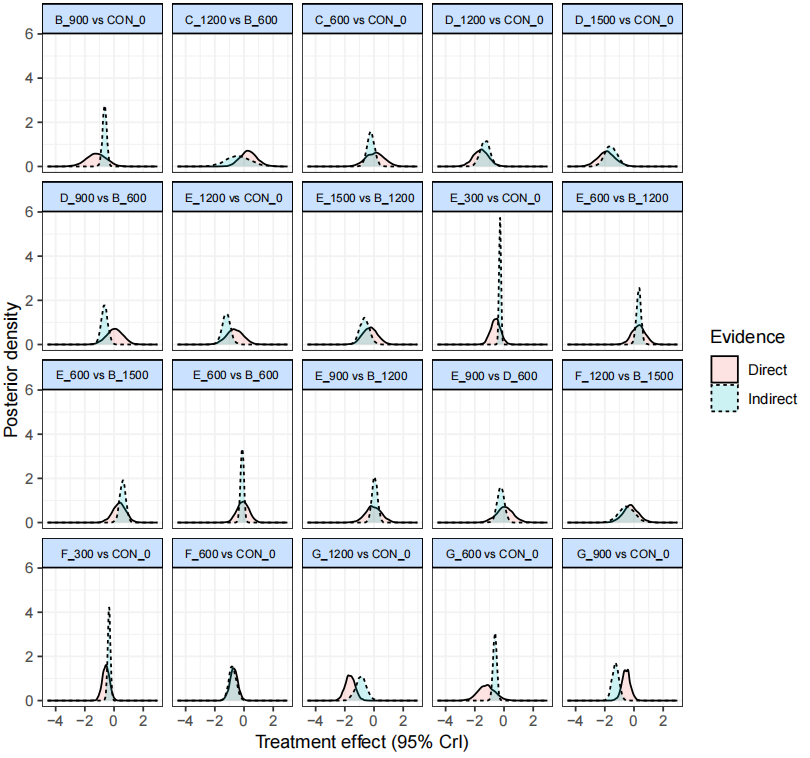


CON: Control; B: Aerobic exercise; C: Resistance exercise; D: Combined exercise; E: HIIT; F: Mind-body exercise; G: Calisthenics.

**Appendix 8** Models’ selection

**1.Non-linear functions and models fit comparison**

A meta-analysis (i.e., a "split" NMA) of the different doses of physical activity as separate and unrelated treatments were performed. This step helps determine which function is more appropriate for the data and should be used in a model-based network meta-analysis (MBNMA)(Mawdsley et al., 2016). Appendix 8-1 and Appendix 8-2 show the different result of each dose to overall and different intensity of exercise, respectively.


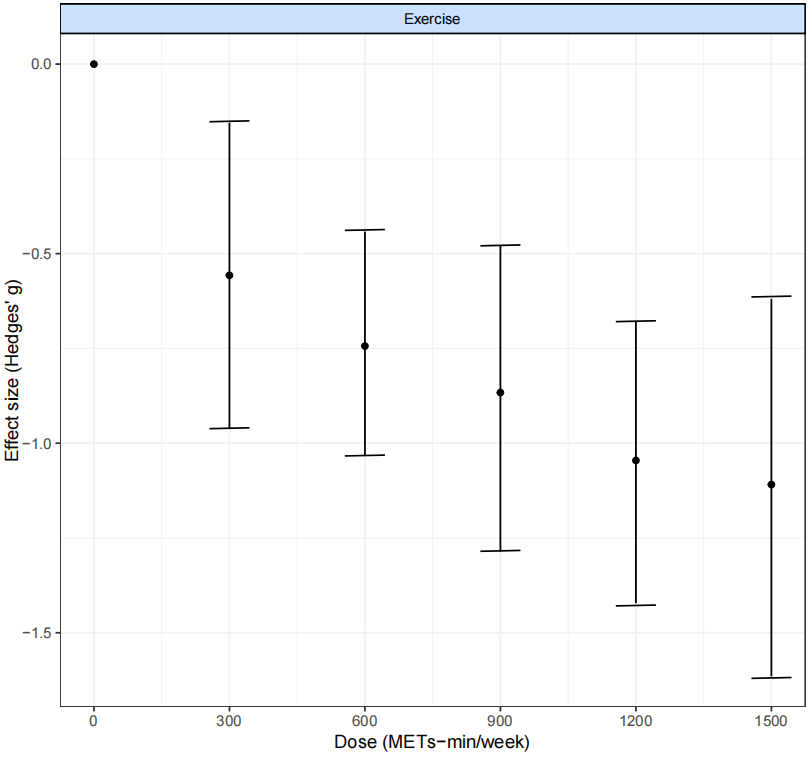


**Appendix 8-1** “Split” NMA of overall exercise.


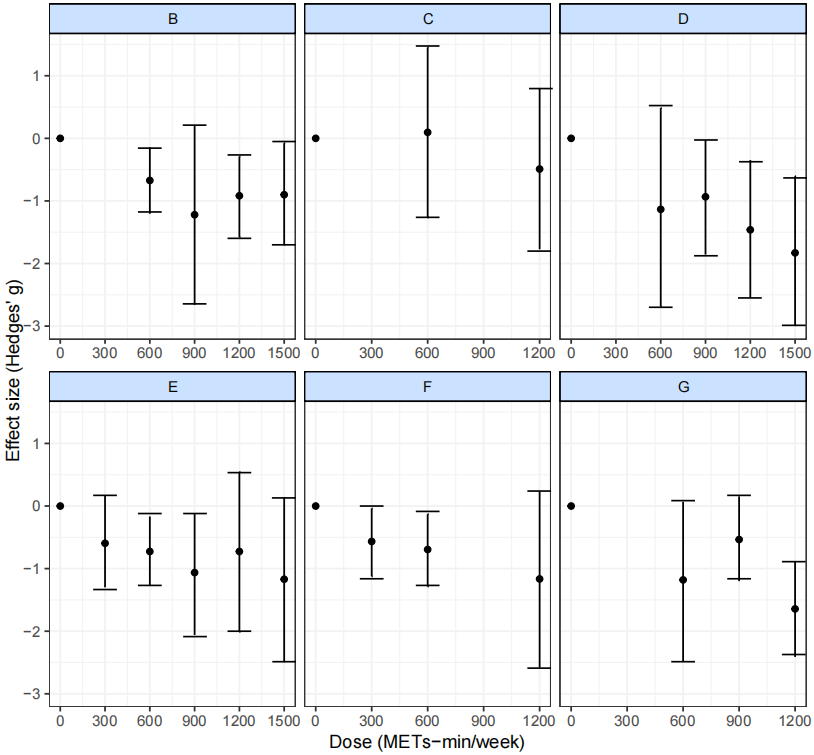


**Appendix 8-2** “Split” NMA of different exercise agents.

B: Aerobic exercise; C: Resistance exercise; D: Combined exercise; E: HIIT; F: Mind-body exercise; G: Calisthenics.

**2.Models selection**

**Appendix 8-3** Models fit comparison

| **Model** | **DIC** | **SD** | **Deviance** | **Residual deviance** | **pD** |
| --- | --- | --- | --- | --- | --- |
| Emax (common treatment effects) | 213.9 | NA | 166.177 | 214.025 | 48.2 |
| Restricted cubic spline (common treatment effects; 3 knots) | 193.2 | NA | 138.289 | 186.138 | 55.8 |
| Restricted cubic spline (random treatment effects; 3 knots) | 117.3 | 0.484(0.338, 0.664) | 39.885 | 87.733 | 78.2 |
| Quadratic function (common treatment effects) | 200.8 | NA | 148.348 | 196.196 | 53 |
| Quadratic function (random treatment effects) | 117.9 | 0.499 (0.353, 0.678) | 39.722 | 87.57 | 78.8 |
| Non-parametric monotonically up (common treatment effects) | 574 | NA | 532.025 | 579.873 | 42.6 |

For our data, quadratic model shows the best fit and were therefore used in subsequent analyses.

**3. Deviation check**

In addition to the model fit index, a deviation plot showing the contribution of each data point to the residuals can also help to confirm the robustness of the model selection. The contribution of each data point to the posterior mean bias should be around 1, which indicates a good model fit(Dias et al., 2013). Deviation plots for overall **(Appendix 8-4)** treatment effects **(Appendix 8-5)** confirm the robustness of our model selection (i.e., deviations <1.5).

**Appendix 8-4** Deviance plot at overall exercise


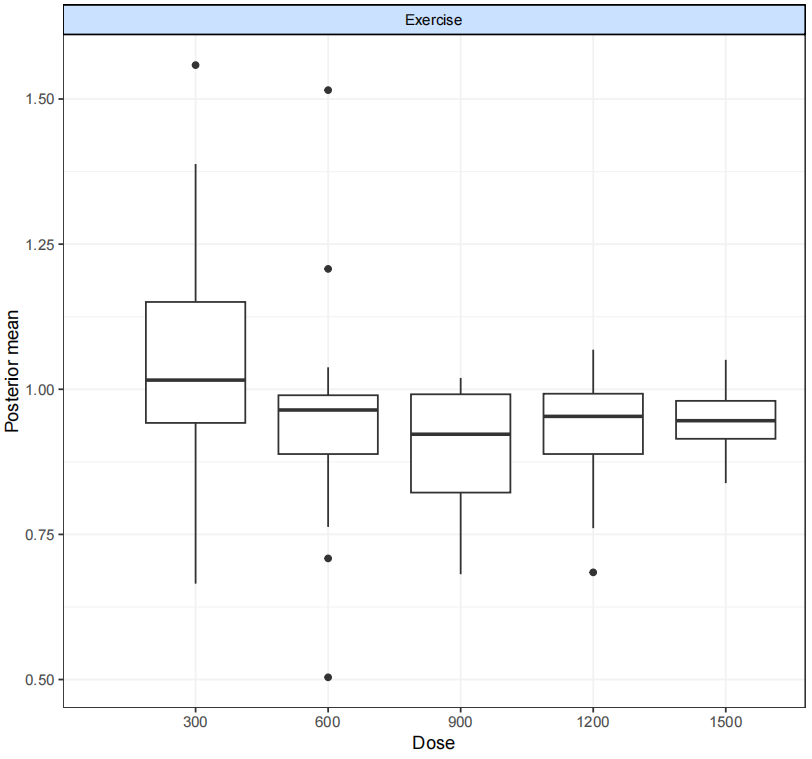


**Appendix 8-5** Deviance plots at treatment-level


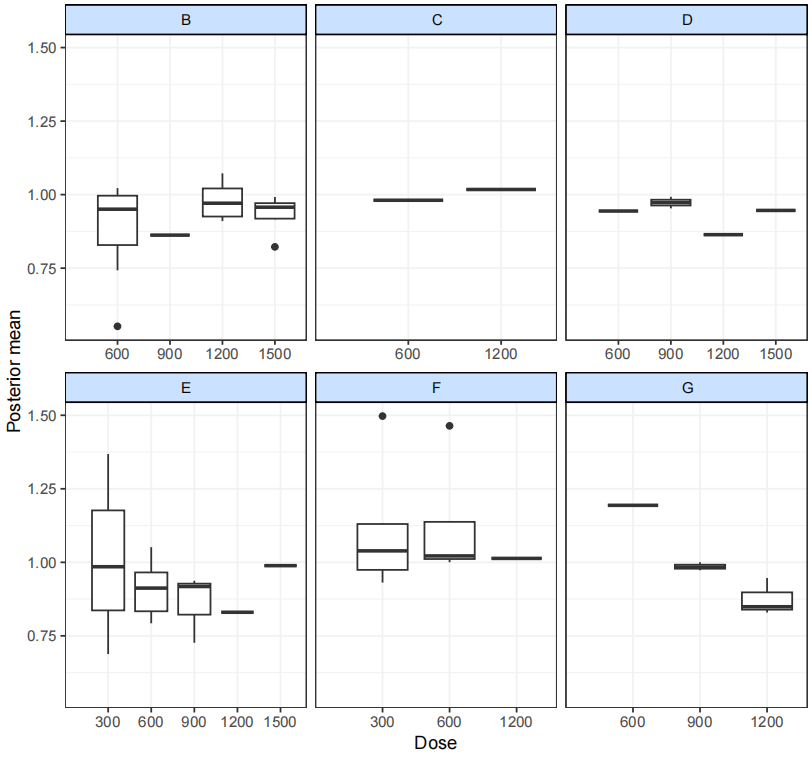


B: Aerobic exercise; C: Resistance exercise; D: Combined exercise; E: HIIT; F: Mind-body exercise; G: Calisthenics.

**4.Degree of fit test**

In addition, we also plotted the fit further to assess the degree of fit of the model. The fit values are plotted as connecting lines, and the observations in the original dataset are plotted as points. These plots can determine if the model fits the data well for different exercises and doses of the dose-response function(“Dose-response functions,” n.d.).

**Appendix 8-6** Fit plots at overall exercise level.


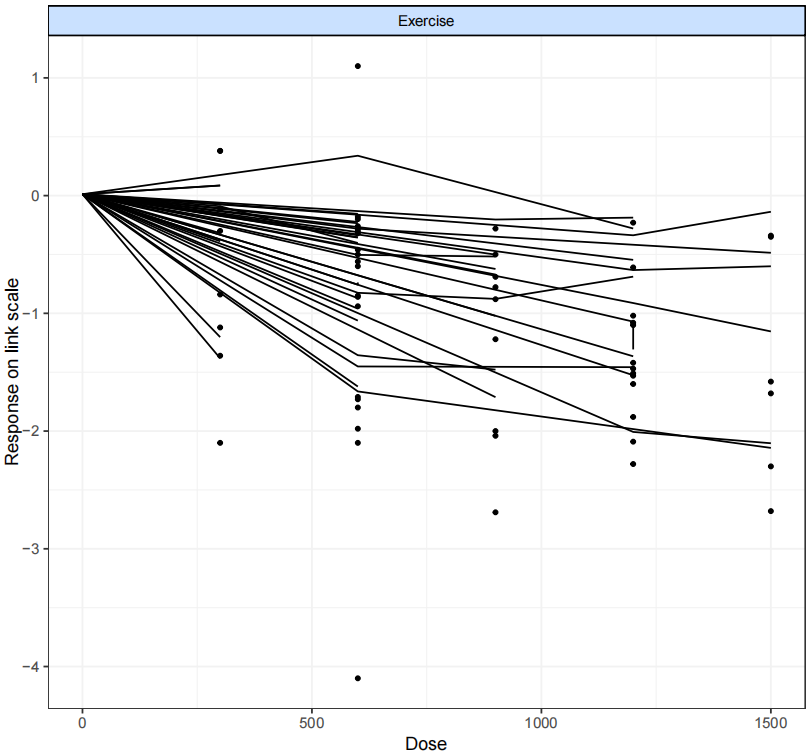


**Appendix 8-7** Fit plots at agent-level.


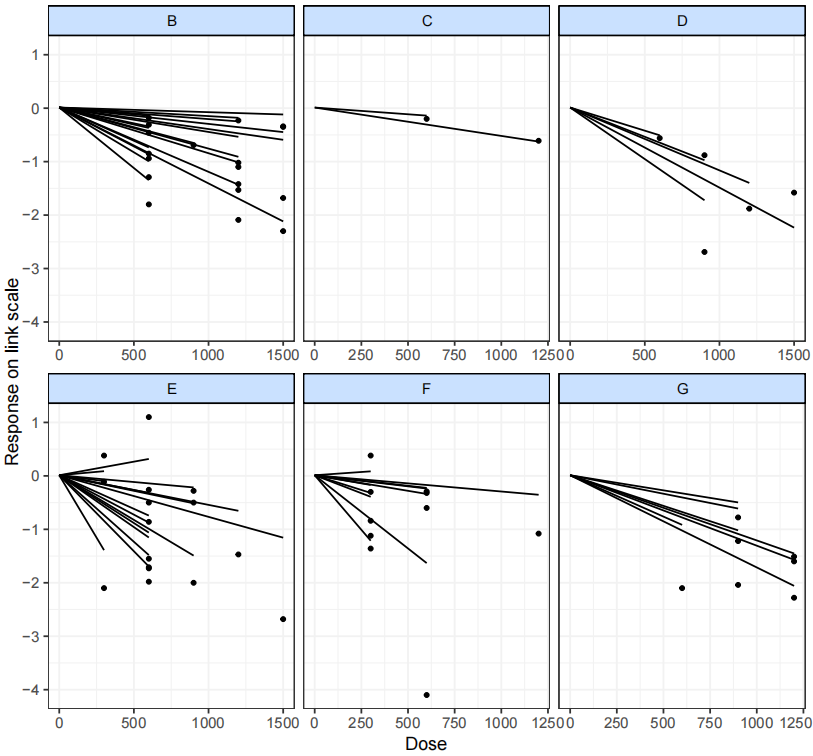


B: Aerobic exercise; C: Resistance exercise; D: Combined exercise; E: HIIT; F: Mind-body exercise; G: Calisthenics.

**Appendix 9** Exercise Dose and BMI Dose-Response Relationships

**Appendix 9-1** Overall Exercise Dose and BMI Dose-Response Relationships


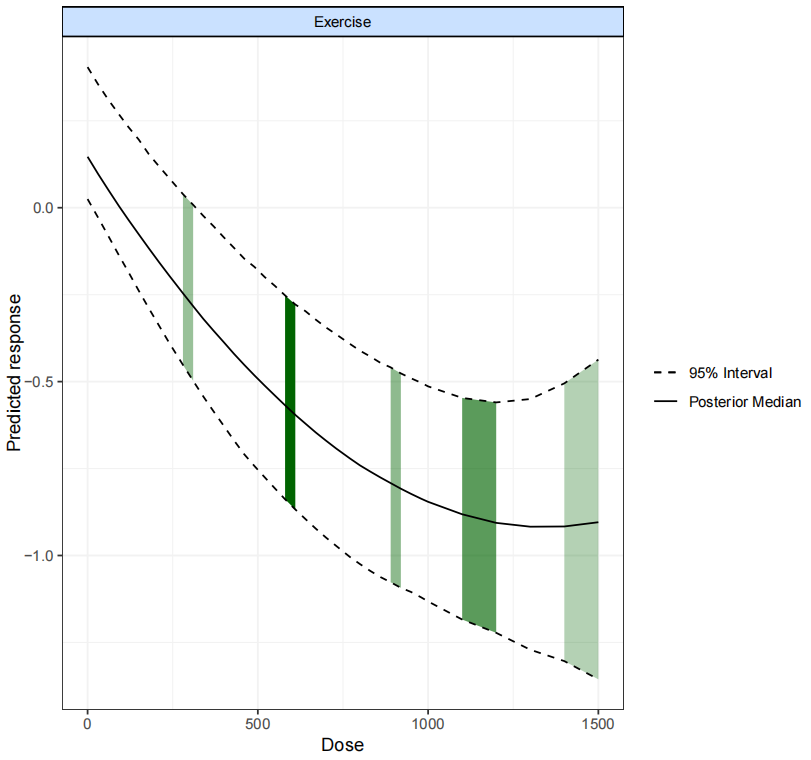


**Appendix 9-2** Dose-Response Relationships of Dose and BMI by Exercise Type


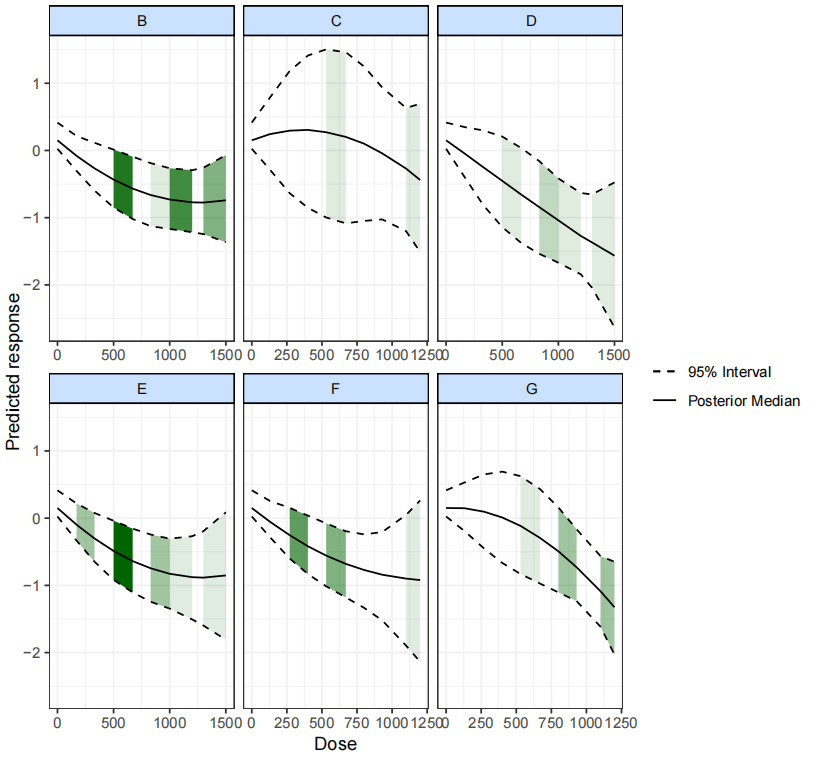


Dose-response relationship between exercise dose and BMI. The green shaded area in the figure indicates the original study dataset; the darker the color, the larger the amount of data.

CON: Control; B: Aerobic exercise; C: Resistance exercise; D: Combined exercise; E: HIIT; F: Mind-body exercise; G: Calisthenics.

**Appendix 10** Ranking the effects of different exercise intensities

**Appendix 10** Sorting Chart of Effects of Different Exercise Intensities


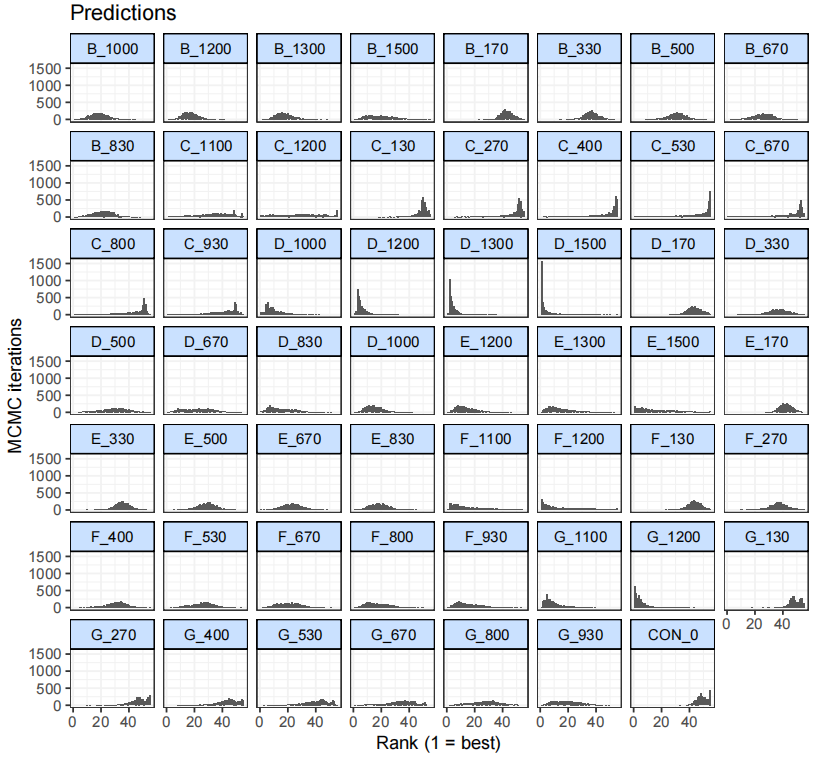


The first value indicates the specific intervention and the second one is the corresponding dose of that intervention (METs-min per week METs-min per week).

CON: Control; B: Aerobic exercise; C: Resistance exercise; D: Combined exercise; E: HIIT; F: Mind-body exercise; G: Calisthenics.

**Appendix 11** GRADE assessment

**11-1. Summary of study limitations of the included studies.**

The colours of the line indicate the summative ROB assessment of each comparison based on ROB assessment of each included studies (low ROB comparison [green], moderate ROB comparison [yellow] and high ROB comparison [red]).


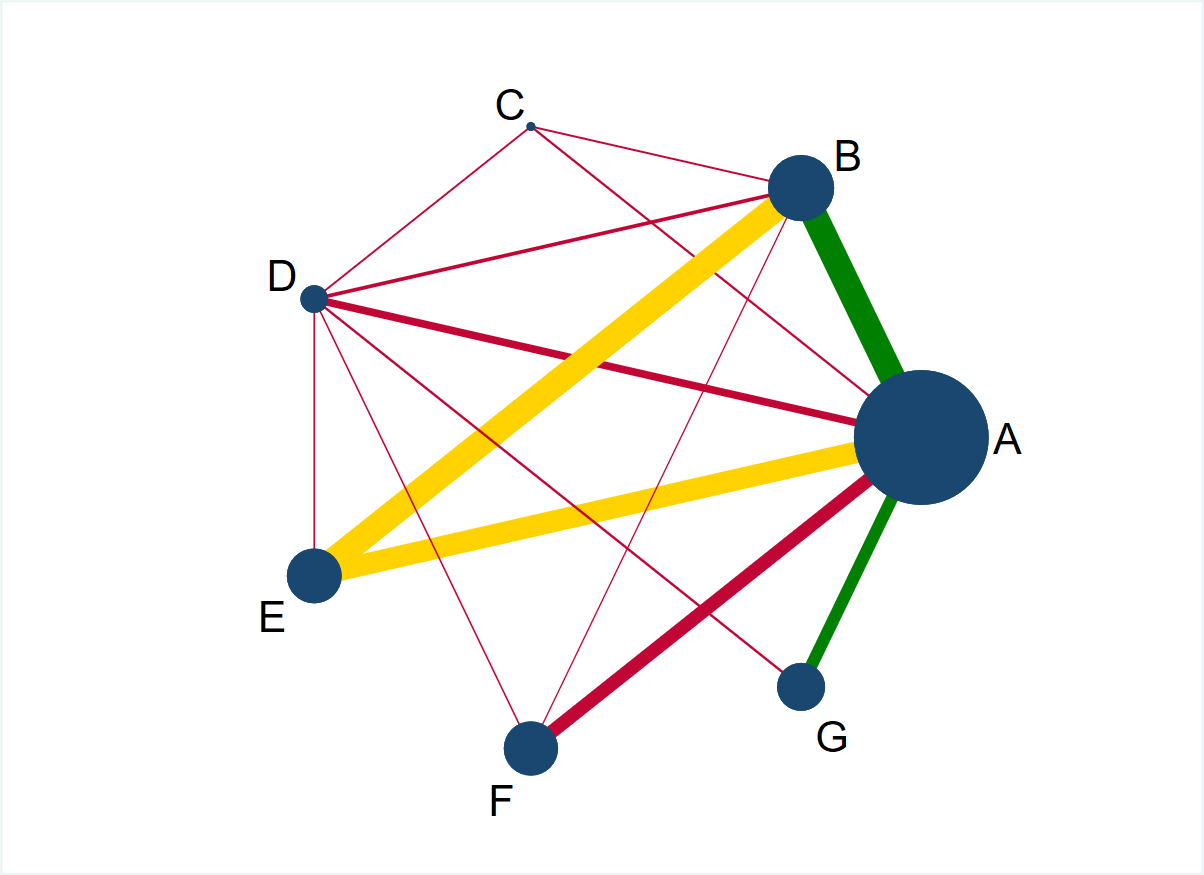


**Appendix 11-1**

A: Control; B: Aerobic exercise; C: Resistance exercise; D: Combined exercise; E: HIIT; F: Mind-body exercise; G: Calisthenics.

**11-2. Contribution of ROB comparisons to each network estimate.**

Based on the above assessment of ROB for each comparison and the contributions of direct and indirect comparisons to all network estimates, the following bar graphs show the percentage of low, moderate and high ROB contributions for each network estimate.


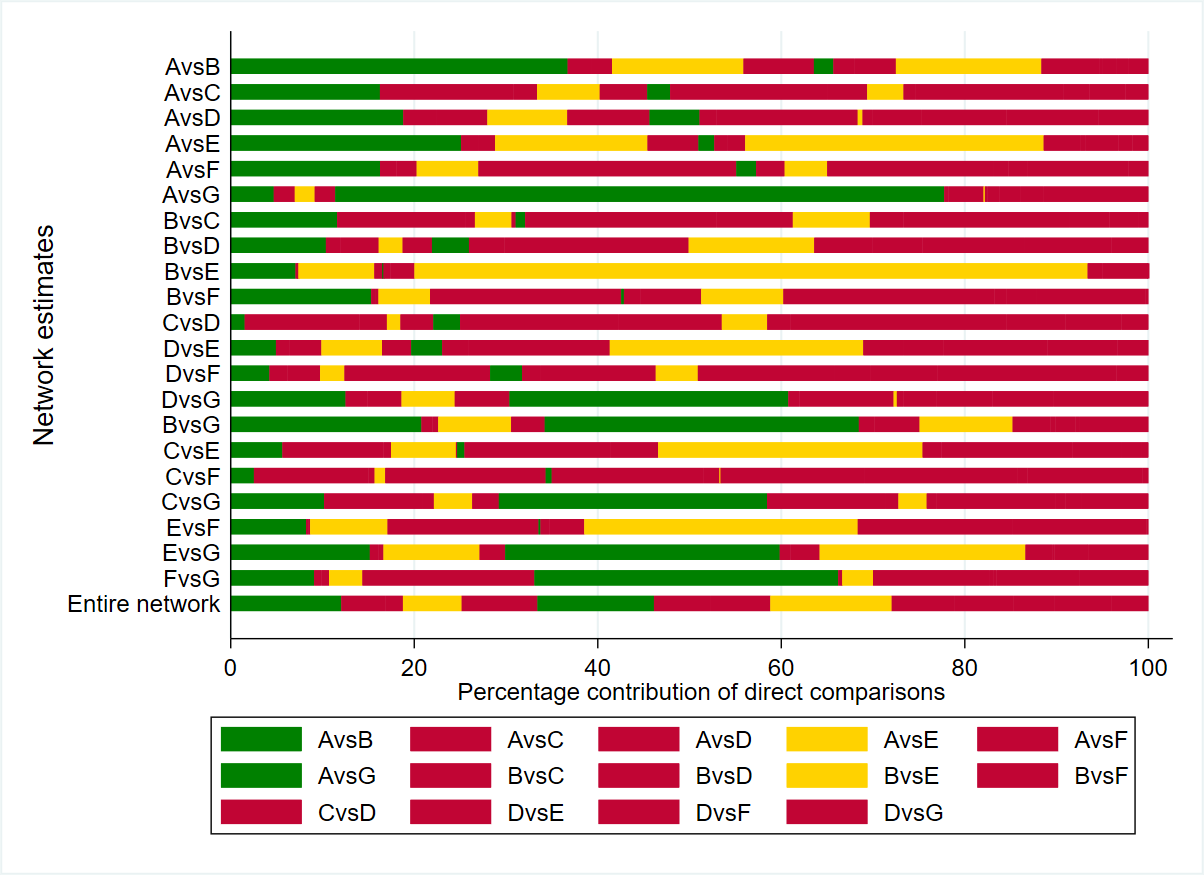


**Appendix 11-1**

A: Control; B: Aerobic exercise; C: Resistance exercise; D: Combined exercise; E: HIIT; F: Mind-body exercise; G: Calisthenics.

**11-3.Table of reasons for downgrading.**

Based on all the above information, we GRADE each network estimate according to the following criteria.

**(1) Study limitations**

Low risk (green)/unclear risk (yellow)/high risk (red) of each direct comparison was given a weight of 0/- 1/-2, respectively. And then the risk value of each comparison and SUCRA probability sorting result were calculated according to the contribution to ROB comparisons to each network estimate. It would be downgraded by one level when the risk value is between -1.5 and -0.6 (indicated by “Study limitations”) and two level when the risk value is less than -1.6 (indicated by “Study limitations^2^”).

**(2) Indirectness**

was assessed by determining whether the study population, type of intervention, and results were directly relevant to the purpose of this meta-analysis.

**(3) Inconsistency**

In this project, we examined heterogeneity and inconsistency. Heterogeneity between each pair of direct comparisons was evaluated by whether the prediction interval of the pairwise forest plots (Appendix 5) crossed the invalid line, it was judged as highly heterogeneous if crossed (indicated by “Heterogeneity”). Inconsistency between direct and indirect comparisons was evaluated by loop-specific heterogeneity estimates and node splitting analysis (indicated by “Inconsistency”). The stability of the SUCRA probability sorting result is evaluated according to global inconsistency.

**(4) Imprecision**

Imprecision was evaluated by appropriate sample sizes, a sample size below 400 would be downgraded by one level. The total sample size in this study was over 400, so this project was not downgraded. The Imprecision of the ranking results is evaluated according to the gap between the ranking values. If the gap between the ranking values of each treatment is small, which indicating a poor stability, the intervention would be downgraded by one leve.

**(5) Publication bias**

Although the NMA comparison-adjusted funnel plot did not suggest presence of overall publication bias, it was subjective evaluation and we cannot completely rule out the possibility that some studies are still missing. Therefore, for the group directly compared with CON, the Begg’s test of pairwise meta-analysis was used to evaluate whether there was publication bias. Considering that the small sample size of groups compared with non-CON may lead to publication bias, all groups compared with non-CON were downgraded by one level.

**Appendix 11-3** GRADE Rating Results.

| **Comparison** | **Nature of the evidence** | **Confidence** | **Downgrading due to** |
| --- | --- | --- | --- |
| AB | Mixed | Low | Study limitations, Heterogeneity |
| AC | Mixed | Low | Study limitations^2^, |
| AD | Mixed | Low | Study limitations, Heterogeneity |
| AE | Mixed | Low | Study limitations, Heterogeneity |
| AF | Mixed | Very low | Study limitations^2^, Heterogeneity |
| AG | Mixed | Low | Study limitations, Heterogeneity |
| BC | Mixed | Very low | Study limitations^2^, Publication bias |
| BD | Mixed | Very low | Study limitations^2^, Publication bias |
| BE | Mixed | Low | Study limitations, Publication bias |
| BF | Mixed | Very low | Study limitations^2^, Publication bias |
| CD | Mixed | Very low | Study limitations^2^, Publication bias |
| DE | Mixed | Low | Study limitations, Publication bias |
| DF | Mixed | Very low | Study limitations^2^, Publication bias |
| DG | Mixed | Low | Study limitations, Publication bias |
| BG | Indirect | Low | Indirectness, Publication bias |
| CE | Indirect | Low | Indirectness, Publication bias |
| CF | Indirect | Low | Indirectness, Publication bias |
| CG | Indirect | Low | Indirectness, Publication bias |
| EF | Indirect | Low | Indirectness, Publication bias |
| EG | Indirect | Low | Indirectness, Publication bias |
| FG | Indirect | Low | Indirectness, Publication bias |
|  |  |  |  |
| Ranking of treatments |  | Moderate | Study limitations |

**References**

Dias, S., Sutton, A.J., Ades, A.E., Welton, N.J., 2013. Evidence synthesis for decision making 2: a generalized linear modeling framework for pairwise and network meta-analysis of randomized controlled trials. Med Decis Making 33, 607–617. https://doi.org/10.1177/0272989X12458724

Donegan, S., Williamson, P., D’Alessandro, U., Tudur Smith, C., 2013. Assessing key assumptions of network meta-analysis: a review of methods. Res Synth Methods 4, 291–323. https://doi.org/10.1002/jrsm.1085

Dose-response functions, n.d.

Mawdsley, D., Bennetts, M., Dias, S., Boucher, M., Welton, N.J., 2016. Model-Based Network Meta-Analysis: A Framework for Evidence Synthesis of Clinical Trial Data. CPT Pharmacometrics Syst Pharmacol 5, 393–401. https://doi.org/10.1002/psp4.12091

Ter Veer, E., van Oijen, M.G.H., van Laarhoven, H.W.M., 2019. The Use of (Network) Meta-Analysis in Clinical Oncology. Front Oncol 9, 822. https://doi.org/10.3389/fonc.2019.00822

van Valkenhoef, G., Dias, S., Ades, A.E., Welton, N.J., 2016. Automated generation of node-splitting models for assessment of inconsistency in network meta-analysis. Res Synth Methods 7, 80–93. https://doi.org/10.1002/jrsm.1167

Watt, J., Tricco, A.C., Straus, S., Veroniki, A.A., Naglie, G., Drucker, A.M., 2019. Research Techniques Made Simple: Network Meta-Analysis. J Invest Dermatol 139, 4-12.e1. https://doi.org/10.1016/j.jid.2018.10.028

Wheeler, D.C., Hickson, D.A., Waller, L.A., 2010. Assessing Local Model Adequacy in Bayesian Hierarchical Models Using the Partitioned Deviance Information Criterion. Comput Stat Data Anal 54, 1657–1671. https://doi.org/10.1016/j.csda.2010.01.025
